# Safe and Healthy Para sport project (SHAPE): a study protocol of a complex intervention within Para sport

**DOI:** 10.1136/bmjsem-2022-001392

**Published:** 2022-08-25

**Authors:** Kristina Fagher, Lovemore Kunorozva, Marelise Badenhorst, Wayne Derman, James Kissick, Evert Verhagen, Osman Hassan Ahmed, Moa Jederström, Neil Heron, Ardavan M Khoshnood, Andressa Silva, Göran Kenttä, Jan Lexell

**Affiliations:** 1 Department of Health Sciences, Rehabilitation Medicine Research Group, Lund University, Lund, Sweden; 2 Institute of Sport and Exercise Medicine, Department of Sport Science, Faculty of Medicine and Health Sciences, Stellenbosch, South Africa; 3 IOC Research Center, Stellenbosch, South Africa; 4 Sports Performance Research Institute New Zealand, School of Sport and Recreation, Auckland University of Technology, Auckland, New Zealand; 5 Department of Family Medicine, Faculty of Medicine, University of Ottawa, Ottawa, Ontario, Canada; 6 Amsterdam Collaboration on Health & Safety in Sports, Department of Public and Occupational Health, Amsterdam Movement Sciences, Amsterdam UMC, University Medical Centers – Vrije Universiteit Amsterdam, Amsterdam, The Netherlands; 7 School of Sport, Health and Exercise Science, University of Portsmouth, Portsmouth, UK; 8 The FA Centre for Disability Football Research, The Football Association, Burton Upon Trent, UK; 9 Athletics Research Center, Department of Health, Medicine and Caring Sciences, Linköping University, Linkoping, Sweden; 10 Centre for Public Health, Queen’s University Belfast, School of Medicine, Keele University, Belfast, UK; 11 Emergency Medicine, Department of Clinical Sciences Lund, Lund University, Skane University Hospital, Lund, Sweden; 12 Sports Training Center, School of Physical Education, Physical Therapy and Occupational Therapy, Sports Department, Universidade Federal de Minas Gerais, Belo Horizonte, Brazil; 13 The Swedish School of Sport and Health Sciences, Stockholm, Sweden; 14 School of Human Kinetics, University of Ottawa, Ottawa, Ontario, Canada

**Keywords:** health promotion, prevention, sporting injuries, disability

## Abstract

Elite Para athletes report a high incidence of sports injuries, illnesses and other health issues. Despite this, there are few prevention programmes in Para sport, and many of the existing prevention programmes are not adapted to Para athletes. To improve the success of preventive measures, it has been suggested that sports safety work should facilitate health promotion, including athlete health education. Therefore, the overarching aim of this project is to evaluate an accessible health promotion web platform as part of a complex intervention that aims to improve knowledge of athlete health in Para sport. In this protocol, the development, future implementation and evaluation of the intervention are described. To inform the implementation and use of such interventions, it is recommended to involve end users in the development and implementation process. Therefore, a participatory design process, including athletes and the sports organisation, was used to develop an accessible health promotion web platform. To evaluate this complex intervention, a process evaluation combining quantitative evaluation assessing causal pathways with qualitative methods assessing multifaceted pathways will be used. The primary outcomes are injury/illness incidence, athlete health parameters, health literacy and user behaviour. A cohort of elite Para athletes (n=150) from Sweden and South Africa will be invited to participate. This project will be the first that aims to improve athlete health in Para sport through pragmatic and accessible health promotion. It is a boundary-crossing project that will be conducted in a real-world sport setting, including athletes with different socioeconomic backgrounds.

**WHAT IS ALREADY KNOWN ON THIS TOPIC**

- Elite Para athletes report a high incidence of sports injuries and illnesses.
- A concern is that many of the existing prevention programmes have not been adapted to Para athletes.
- To improve the success of preventive measures, it is suggested that sports safety work should advantageously facilitate health promotion, including athlete health education.

**WHAT THIS STUDY ADDS**

- This project aims to improve athlete health in an understudied and underprioritised population.

**HOW THIS STUDY MIGHT AFFECT RESEARCH, PRACTICE OR POLICY**

- Using a complex intervention design, including the end users, medical experts and researchers in the design process, facilitates transparent information about the development, implementation, feasibility and evaluation, allowing researchers to replicate and improve the method in future studies.

## Introduction

Elite Para athletes report a high incidence of sports injuries and illnesses, and other health issues such as poor sleep and mental illness.^1–3^ Compared with able-bodied athletes, an injury may be even more concerning in Para athletes, due to their existing impairment. For example, a shoulder injury affecting an athlete using a wheelchair can be devastating both in the athlete’s sporting career but also in activities of daily living. In Para athletes with visual impairment (VI), the risk for traumatic injuries is high.^2^ Para athletes also have specific risk factors for illness, for example, athletes with neurological injury may be predisposed to urinary tract infections and pressure sores.^2^

Despite these factors, there are few evidence-based prevention programmes in Para sport, and there is an urgent need to prevent injuries and illnesses and to improve health in Para athletes. Successful injury prevention programmes in able-bodied sports have focused on organised neuromuscular training, strength, plyometrics, flexibility, balance and agility.^4^ A concern is that many existing prevention programmes, such as those aimed at knee control or hamstrings injury prevention, have not been adapted to Para athletes, as many athletes are wheelchair users or amputees.^2^ Another concern is that most prevention programmes focus mainly on reducing the risk of injuries, whereas data show that illnesses are an important concern in Para athletes.^2^

Additionally, the context in which Para athletes participate in sport differs from able-bodied athletes.^5^ The support structures are less organised and many athletes train without input from a coach.^5^ Access to medical services is poor and coaches have limited knowledge of Para athletes and their impairments.^5^ These factors are further compounded by the existing implementation challenges of training-based prevention programmes in real-world sport settings.^6 7^ Recent research has also shown that both athletes and coaches prioritise performance enhancement rather than injury prevention.^8^ To allow athletes to also improve performance, it could thus be suggested that one needs to think beyond specific reactive training-based injury prevention, and focus on disease prevention and health promotion. These prevention and health promotion measures should include athlete health education and interventions aimed at health behavioural change.^9–12^

More specifically, health promotion enables individuals to self-educate themselves on determinants of health and how to improve their health and in their operating context, in this case sport.^13 14^ To succeed with this, the WHO recommends access to quality health literacy education and health guidance.^14^ For Para athletes, it could be hypothesised that such information is important as it is known that persons with a disability have poorer health and lower health literacy than persons without disability.^15^ One way to disseminate such information is through eHealth, which is the use of information and communication technologies for health.^2^ However, many eHealth measures fail to reach persons with a disability due to non-adapted interventions that are not physically or technically accessible.^14^ To improve health literacy among persons with disabilities, it has been recommended to focus on clear, accessible and effective health communication advocating physical and mental health, and equity.^13 15^

For athlete health research to move forward, it has also been recommended that each sport’s context and complexity be considered in developing preventive measures.^7^ Moreover, it is important to define the health problems in the specific athlete population and to understand the possibilities for the individual athlete to perform and adopt the proposed prevention measures.^7 8^ This is crucial among Para athletes, as these athletes have different impairments and abilities. Many gaps in the implementation of preventive measures in sports are also due to failures in the reach and adoption by sports organisations.^16^ Indeed, it has been proposed that policies, attitudes and involvement of sports organisations in the development of sports safety are of utmost importance.^6 7 17^ Thus, to improve the success of preventive measures, the development of sports safety initiatives should be considered as complex interventions, which takes all levels of a hierarchical sports system into account, including the athlete, coaches and the sports organisation.^10 11 17^

The overarching aim of this project is to develop, implement and evaluate an accessible health promotion web platform in a complex intervention that aims to improve knowledge of athlete health in Para sport. The specific aims that are described in this study protocol are: (1) to develop an eHealth-based and accessible health promotion web platform for Para athletes and their coaches, (2) to identify contextual factors within the sports and organisation that can facilitate the fidelity, implementation and use of such an intervention, (3) to assess whether a health promotion platform can reduce sports injuries and illnesses in elite Para athletes, (4) to assess whether such a platform can improve overall health parameters, such as sleep, nutrition and mental health in elite Para athletes and (5) to assess whether such a platform improves health literacy and changes behaviour among elite Para athletes.

## Methodology

**Figure 1:**
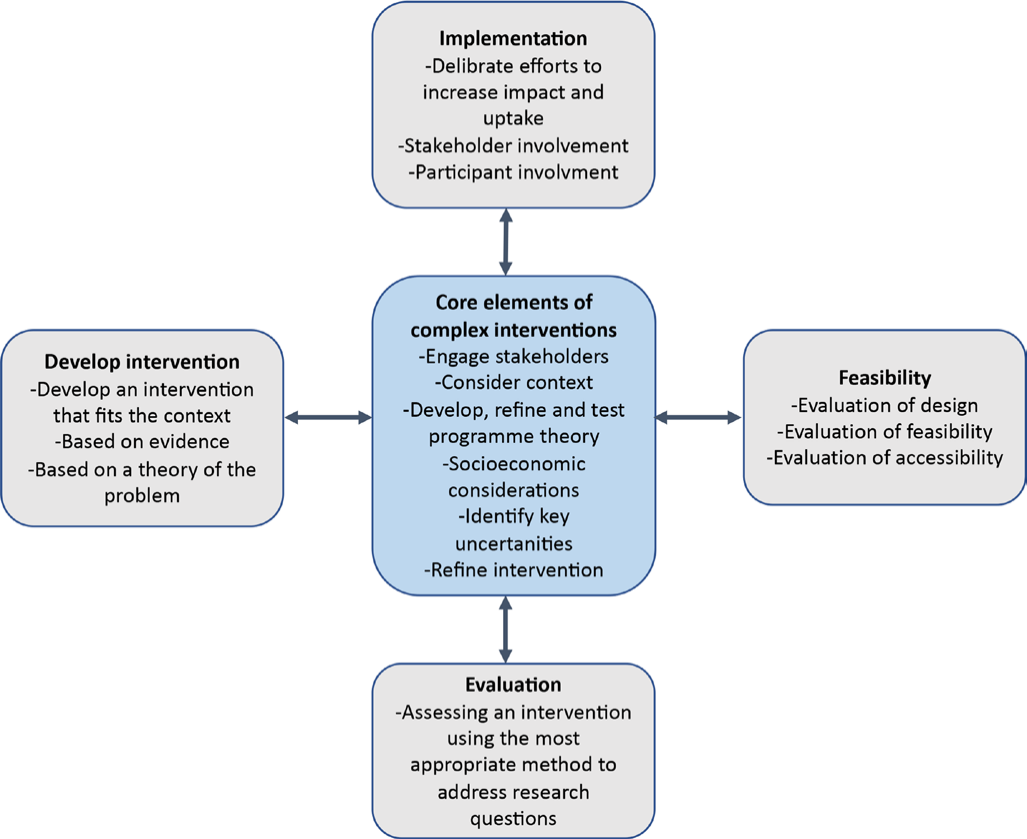
A framework for development, implementation and evaluation of a complex intervention described by Skivington *et al*^18^ in Medical Research Council’s updated guidance for complex interventions and modified for this project.

**Figure 2:**
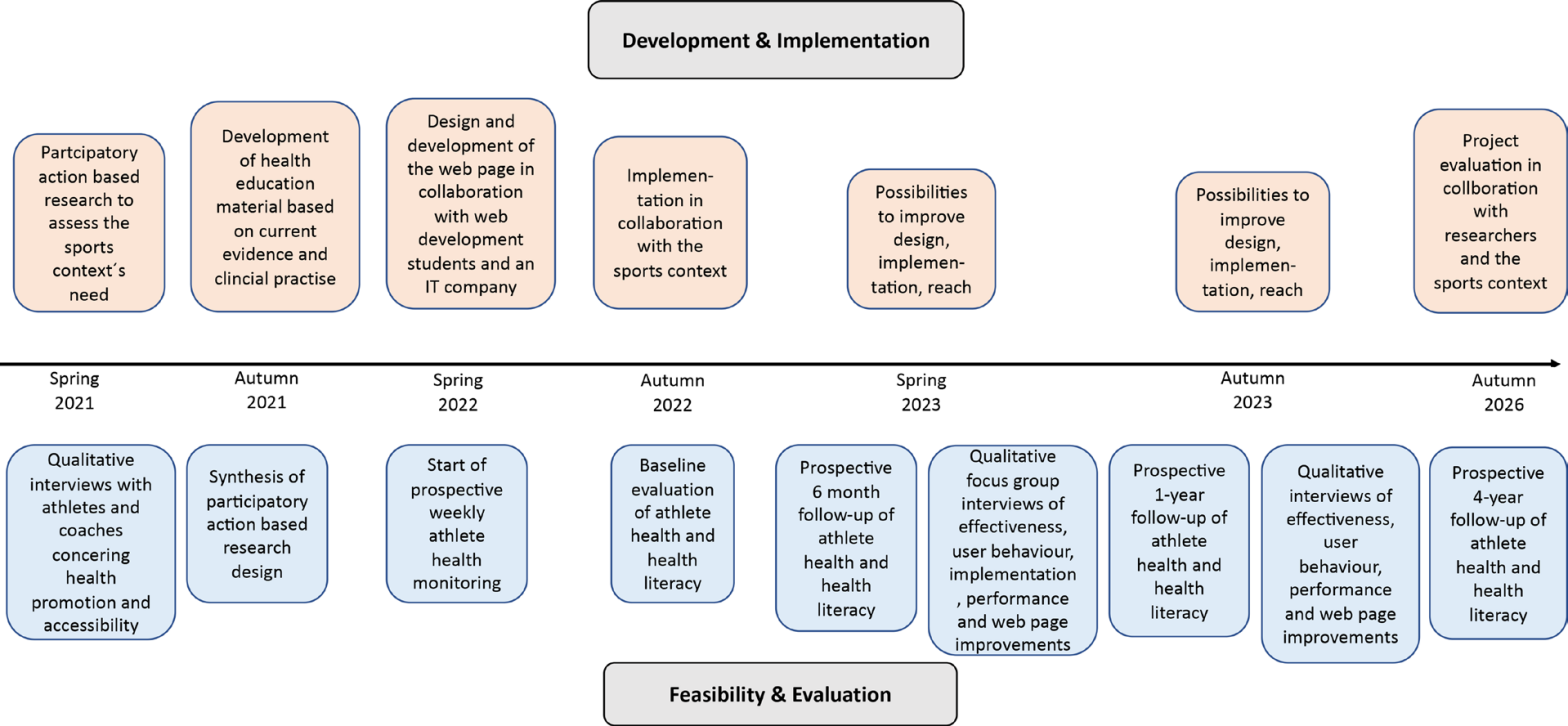
Flowchart of the development, implementation, feasibility and evaluation of the complex intervention Safe and Healthy Para sport.

Complex intervention research goes beyond evaluation of a single intervention in a fixed and controlled research context.^18 19^ The method emphasises a pluralistic approach to evaluate how an intervention should be developed, delivered and implemented to test the intervention’s feasibility in a real-world context and determine how such an intervention can contribute to change.^18 19^ A complex intervention design is especially feasible when a heterogeneous population is being studied, particularly when behavioural factors are included.^18^ The development, implementation and evaluation of this complex intervention are described in more detail below and in figures 1 and 2.

### Development of the health promotion platform

**Figure 3:**
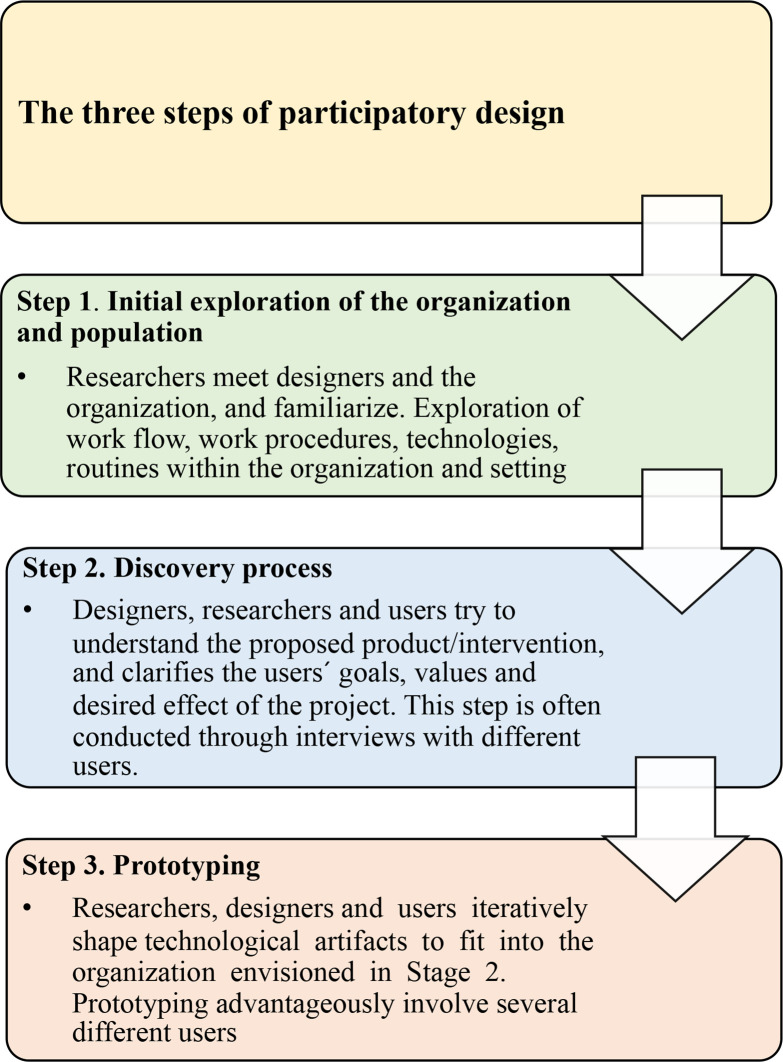
A description of the participatory design method that was used to develop the health promotion platform.

To develop the eHealth-based health promotion platform ‘Safe and Healthy Para sport’, participatory design (PD) based on intelligent user interface adaption was applied.^20 21^ PD is a research method that emphasises that developers, researchers and users actively collaborate in a process aimed to improve the quality of life for a specific population. The PD includes three steps, and the sections below describes how aims (1) and (2) have been addressed (figure 3).

### Participant and public involvement

The main idea with the iterative PD process is to allow the users (athletes, coaches and the organisation) to influence the development and decisions by sharing knowledge and experiences.^20 21^ In this study, participants that were involved in the PD process were: (1) researchers with experience in sports and exercise medicine (SEM) and Para sport, (2) the performance team of the Swedish Paralympic Committee (SPC), (3) the media team of SPC, (4) external IT and accessibility consultants/students, (5) athletes with various impairments, ages and gender and (6) coaches with various background, gender and ages.

### Step 1: initial exploration of the organisation and population

The first step in the PD process, the development phase, is to form a design group consisting of researchers and developers and initially explore an organisation’s workflow and expectations. The design group (KF, LK, MB, WD and JL) consists of researchers from Sweden (high resourced nation) and South Africa (lower resourced nation), high performance directors of SPK, the education director of SPK and IT developers. The group had several meetings during the spring 2021 to discuss the organisation’s needs, workflow, technologies, expectations and routines^20 21^ (figures 2 and 3).

### Step 2: discovery process

The discovery process, which is the second step of the PD design, enables researchers to try to understand and clarify the users’ expectations, values and desired effect of the intervention through participant engagement. Participant engagement has also been shown to improve study design, promote recruitment and make the participants more amenable for uptake of the intervention.^22^ This step is often conducted through interviews,^20 21^ which is supported by current research in SEM.^8^ Hence, participant engagement interviews using a qualitative thematic analysis was conducted in 2021. Thematic analysis is a flexible method for identifying and analysing patterns and needs when developing interventions.^23^

Individual interviews were conducted by three members of the research team (KF, LK and MB). A semi-structured interview guide was used and athletes’ and coaches’ were asked about their perspectives on: (1) common health problems in Para sport, (2) the possibilities to prevent injuries and illnesses in Para sport, (3) health promotion in Para sport and (4) the expectations of such intervention in terms of content, accessibility and adoption. The data were condensed and categorised into themes, codes and meaning units.

A purposive sampling was used to ensure variation in impairment and sports, and in total seven male and six female elite athletes from Sweden and South Africa were interviewed. The athletes had the following impairments: physical impairment (n=8), vision impairment (n=3) and intellectual impairment (n=2), and represented the following Para sports: boccia, goalball, judo, swimming, cycling, athletics, wheelchair curling, para ice hockey and cross country skiing. The mean time spent on training each week was 16.8 hours/week, and the athletes had on average been active in Para sport for 14.8 years. In addition, three male and three female coaches from Sweden and South Africa were interviewed. Their mean experience of coaching in Para sport was 18.2 years. The coaches were active in the following Para sports: table tennis, shooting, cycling, judo, athletics and Para alpine skiing.

### Results of participant engagement interviews

The results revealed four high-level themes: Para sport-specific injuries and illnesses, prevention, health promotion, and adaptation and implementation (table 1).

**Table 1:** Thematic analysis of Para athletes’ and coaches’ perceptions of common injuries/illnesses and risk factors of sports injuries and illnesses in Para sport, the possibility to prevent injuries, health promotion and expectations of a possible health promotion intervention

Theme	Codes	Meaning unit
Para sport-specific injuries and illnesses	Injuries occur in Para sport Impairment-specific risk factors General health	All athletes have had a sports injury Take long time to heal Infections are concern Comorbidity and daily life activity strains
Prevention	Adapted prevention measures Individual prevention measures	Most prevention measures are not adapted Need for prevention Coaches should have knowledge Different impairments and prerequisites
Health promotion	Para sport-specific health education	Need for better understanding of athlete health and the impairment Need for better understanding of training and the impairment Need for better understanding of factors such as sleep, nutrition and mental health
Adaptation and implementation	Accessibility The sports context	Must be adapted to athletes with various impairments Reminders within the sports and social media The educational web platform should be implemented within the sports context

#### Para sport-specific injuries and illnesses

The participants described that sports injuries are common in Para sport. One national team coach described how all his athletes had had an injury, and that many of these injuries were linked to the impairment. A perception shared by athletes and coaches was that many overuse-related concerns arose in the athletes’ daily life due to factors such as unfavourable load and inadequate recovery. For example, the participants expressed that athletes using a wheelchair are prone to shoulder injuries, while athletes with cerebral palsy are prone to muscle imbalance. Several athletes expressed that they are also exposed to the same injuries as able-bodied athletes such as ligament injuries and that the rehabilitation is complicated due to factors such as spasticity and impaired mobility.

*"I had pain in my shoulder, it was bone to bone and the rotator cuff was torn. I believe it was caused because of what is happening in the lower part of my body." (Athlete 5)*

Another perception was that injuries take a long time to heal in Para athletes due to inadequate access to healthcare and rehabilitation. Concerning illnesses, most participants perceived that infections are a concern for elite athletes. The athletes’ perceptions were that they have the same risk for infections as able-bodied athletes, whereas coaches perceived that impairment-related factors and poor health increased this infection risk. Coaches also reported that the general health in Para athletes is inferior compared with able-bodied athletes, which was explained by impairment-related comorbidity and strains in daily life. For example, both athletes and coaches expressed that it can be challenging for athletes with VI and intellectual impairment to plan activities such as nutrition, recovery and training when being an elite athlete.

In summary, health problems in Para athletes are multifactorial, including injuries, illnesses and impairment-related comorbidities, which needs to be considered in health promotion in Para sport.

#### Prevention

The participants’ perceptions were that there is a need for implementing preventive measures in Para athletes, as many of these athletes regularly suffer from injuries, illnesses and adverse mental health. A perception was that many prevention measures in sports are developed for able-bodied athletes, and do not consider Para sport-specific factors. For example, one athlete with VI expressed that concussion prevention initiatives should consider that the athlete has a vision impairment. The participants requested more Para sport-specific prevention in the future. Another concern that several participants described is that there is a need for individual prevention measures, as Para athletes have different impairments and prerequisites.

*"For me as a VI athlete, injury prevention is not only about specific exercises, but also about having a guide, that my peers know that I am blind, that all obstacles are removed and that I am feeling recovered mentally." (Athlete 2)*

An athlete with intellectual impairment voiced the importance of coaches’ knowledge about both the impairment and prevention measures to optimally support athletes with this impairment. Collectively, this theme illustrates the need for Para sport-specific prevention.

#### Health promotion

Several athletes reported that many coaches did not have knowledge about Para athletes’ health, and the athletes requested more specific education for coaches. For example, one athlete with spinal cord injury (SCI) expressed that coaches did not have any knowledge about bladder and urinary tract problems in SCI athletes. Coaches expressed that it is challenging when Para athletes train with able-bodied athletes, without the clubs and coaches having knowledge about athletes’ impairments.

*"A concern is that new coaches within Para sport quite often lack knowledge about the impairment, and, therefore, they push the athletes too hard or too little." (Coach 3)*

One coach also raised the importance of athletes themselves having knowledge about training and injury prevention to become a successful Para athlete. Furthermore, the athletes themselves wanted to learn more about ways to improve their health and prevent injuries and illnesses, and a majority of the participants felt that education could help them to improve. For example, one athlete described that he wanted to learn more about prevention of pressure ulcers, whereas one athlete did not know about SCI and endurance training. A coach expressed that it is important to have knowledge regarding nutrition and hydration. Other important topics mentioned by the participants were training-related considerations in female athletes, mental health and travelling.

Taken together, the perceptions were that there is a need for more knowledge and education about athlete health and training in Para sport.

#### Adaptation and implementation

Concerning the athletes’ impairments, the participants revealed that health promotion interventions must be accessible. For example, an athlete with intellectual impairment described the need for short text, large text and easy text, whereas athletes with VI described the importance of reading text aloud, adapting text size and avoiding too many pictures. Overall, both athletes and coaches expressed that a health promotion intervention in Para sport should not include excessive or voluminous text.

The athletes suggested promotion in social media and regular reminders to inform implementation and use. Coaches’ perceptions were that a potential web platform must be implemented and promoted within the sports context to reach both athletes and coaches.

*"The platform must be promoted within the sports, otherwise no one will use it." (Coach 6)*

It was recommended to organise seminars about the intervention at both organisational level and within the sports context, for athletes and coaches, and that the sports context itself should regularly remind the target population about the intervention.

Taken together, a health promotion intervention in Para sport should be accessible and implemented within the sports context.

### Step 3: prototype development

**Figure 4:**
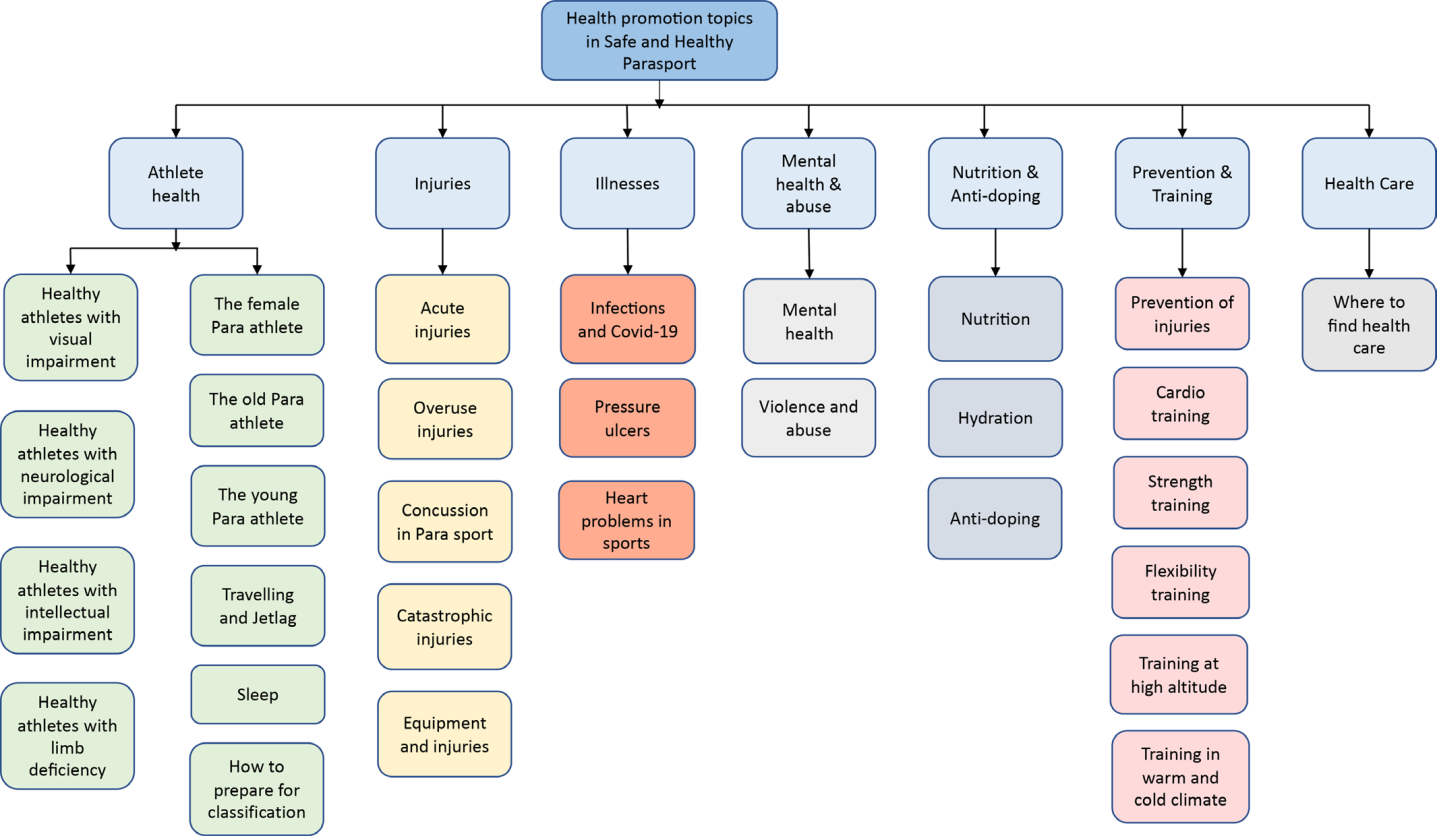
Health promotion topics that will be presented at the health promotion webpage: Safe and Healthy Para sport.

In the third and last step within the PD design, the researchers, organisation, developers and users iteratively shape the prototype. Based on the results from the previous steps, including discussions with Para sport research experts, the sports context and participant engagement interviews the design group (KF, LK, MB, WD and JL) decided that the platform’s content would contain the following health promotion topics: athlete health, injuries, illnesses, mental health and abuse, nutrition and antidoping, prevention and training and healthcare (figure 4), which is similar to the already established web platform ‘Medicine and Health in Athletics’.^24^ Each section will focus on Para sport-specific information, and the health promotion content will be established based on the best-identified current evidence and/or best practice according to a health service guideline development procedure.^25^ During the autumn 2021, 20 international experts were invited to write the material.

Another crucial part that the PD design revealed was accessibility. To develop the webpage technically and ensure accessibility based on the Web Content Accessibility Guidelines,^26^ contact was established with Blekinge Technical University, Sweden (www.bth.se). A student group consisting of five web design students then collaborated with the IT company WIP (www.wip.se) to develop and adapt the webpage. Based on the previous results, a requirement specification was first formed (table 2). To ensure feasibility, two athletes with vision impairment, one athlete with intellectual impairment and one athlete with severe physical impairment were asked to regularly evaluate the functionality of the webpage during the design process.

**Table 2:** A requirement specification and product adaptation of a health promotion webpage adapted to para athletes based on a participatory research design process

Functional requirements	Front-end criteria	Back-end criteria
Functionality of a wide varity according to the standard set by WCAG	Adaptations to persons with visual impairment: choose colour of website to match with colourblindness and light sensitivity Speech synthesis function Need to resize fonts/text for better visibility Need to be able to remove images, or place them in the end of a text and have them to have a description Avoidance of too many tabs Adaptations to persons with physical impairment: Control the webpage using keyboard actions Text that can be read aloud Adaptations to persons with intellectual impairment: Indication of reading level Understandable text Indication of leaving a page Understand how to get to the page I want Picture and videos for visualisation Text that can be read aloud	Application changing colour scheme Website to work well with JAWS and Supernova screen readers Application changing text size Layout and audio description Design Keyboard application Readable text application Design and information Design and information Webpage exit application Colour marked links Design and layout Insertion of videos and pictures Readable text application
Functional on ‘most’ devices	Able to view the webpage on computers, tablets and phones	Multidevice site
Article viewing	Categories and tabs Search for articles/topics	Layout and design Search application
A webpage design appealing to the demographic	Social media feed Able to switch between Swedish and English version Targeting athletes in the lower middle age	Social media application Language application Layout and design
Administration requirements	Edit articles Keep track of users	User friendly admin page User track application

### Translation of research to practice

To plan, evaluate and describe the translation of this scientific project into practice, the RE-AIM planning and evaluation framework will be used.^27 28^ This model, that includes the following steps: reach, effectiveness, adoption, implementation and maintenance. It has an underpinning of health promotion theory, suggesting that desired behaviours will only be achieved if interventions are available to the target group, adopted by them and used as they were intended to.^28 29^ Moreover, it has been suggested that RE-AIM can be used to document adaptations of an intervention both prior, during and after the implementation. The framework highlights the need for stakeholder buy-in, and, therefore, the implementation process started at an early stage in this project.^27^

For the first step, reach, it is recommended that the target population understand and participate in developing an intervention. Relationships were, therefore, built early with the community and target population through project presentations, regular meetings and qualitative interviews (as described in steps 1 and 2 in this paper). To reach and evaluate the effectiveness of health promotion interventions, it is important to evaluate the impact of an intervention on both the context and individual level. Furthermore, it is important to analyse health behaviour, potential negative and positive effects, and intervention improvements. In a long-term perspective, it is also crucial to determine maintenance of the intervention. As such, effectiveness will be evaluated by longitudinally assessing athlete health and health literacy, and by qualitatively assessing user behaviour and intervention improvements (figure 2).

Adoption is the proportion and representativity of a setting and intervention agents willing to initiate and deliver a programme. To further understand the adoption, the organisation, coaches and researchers were and will be included in the development and design of the intervention as described in this protocol.

At the setting level, implementation refers to the intervention agents’ fidelity to the various elements of an intervention’s key functions or components, including consistency of delivery as intended, and the time and cost of the intervention. It also includes adaptations made to interventions and implementation strategies. To inform implementation within this project, the end users were involved early in the design process. Stakeholder interviews will be conducted with the SPK and the Maties Para Sport Club, Stellenbosch, in South Africa plan and maximise the intervention. The complex intervention design will also allow us to improve the implementation during the first 6 months and 1 year.

Maintenance is how an intervention becomes part of the routine organisational practices and policies. Within the RE-AIM framework, maintenance also applies at the individual level. At the individual level, maintenance has been defined as the long-term effects of a programme on outcomes after a programme is completed. A qualitative focus group study will be conducted after 6 months with athletes, coaches and stakeholders to assess maintenance. After 1 year, qualitative interviews will be conducted with the same population to assess user behaviour (figure 2). To further monitor the adoption and maintenance, regular analyses of user statistics from the webpage will also be conducted and a final project evaluation will be conducted 2026.

### Process evaluation

To evaluate this complex intervention (aims 3–5), a process evaluation will be used. Process evaluation is a method that can be used when developing, implementing and evaluating complex interventions within a specific context. The method combines quantitative evaluation assessing causal pathways with qualitative methods assessing complex pathways and unexpected mechanisms.^19^ The UK Medical Research Council Guidelines have recognised that process evaluations can assess both the implementation and fidelity of an intervention and identify contextual mechanisms and outcome factors that are specific to the population and context they operate in.^18 19^

The process evaluation in this study will include a quantitative longitudinal prospective evaluation of athlete health outcomes, a cross-sectional and prospective evaluation of health literacy and qualitative evaluation of the development, implementation and effects of the intervention (figure 1). Six months before implementing the web-based platform, all (approximately 150) athletes from the SPKs and the Maties Sports Club, South Africa, will be invited to participate in a longitudinal athlete health surveillance study. Due to a total population design, there is no power calculation. All athletes will be asked to self-report the health outcomes of injuries, illnesses, mental health, sleep, nutrition, time loss from training and training quantity and quality weekly, using the eHealth-based and adapted surveillance system that has been used and evaluated in a previous prospective study.^1^

At the end of 2022, the web platform ‘Safe and Healthy Para sport’ will be implemented online as an open web platform webpage. All athletes will be encouraged to use this web platform each week to improve their overall health knowledge, specifically when experiencing symptoms of an injury, illness or other health concern. The athletes will receive a weekly invitation/reminder about a specific topic to learn more about. Each week’s health topic will also be presented on the project’s social media platforms. The primary outcome is the incidence of injuries and illnesses. Secondary outcomes are the health parameters sleep quantity, nutrition, mental health and the performance indicators training quality and quantity. Data will be analysed by describing incidence rates and proportions. A time-to-event analyses will be conducted. Data will be collected and analysed 6 months before implementation, 6 months after implementation and at 1 year and 4 years following implementation (figure 1).

To evaluate whether the platform has had any impact on health literacy, all athletes will be asked to complete the Literacy in Musculoskeletal Problems,^30^ and the Health Literacy Survey European Questionnaire^31^ 1 week before the implementation of the webpage, and 6 months and 1 year following the implementation of the webpage (figure 2).

Focus group interviews concerning the delivery of the webpage, implementation, accessibility, feasibility, the health topics, use of the webpage and possible improvements will be conducted with athletes, coaches and stakeholders 6 months after the implementation. Following this evaluation, adjustments may be applied. Individual qualitative interviews with athletes, coaches and stakeholders will be conducted 1 year after implementation about the effect of the web platform, user behaviour and potential for improvement. Furthermore, it will be evaluated whether the uptake and effectiveness of the intervention is similar in the two countries (Sweden and South Africa) with different socioeconomic contexts.

## Discussion

This project will be the first that aims to improve athlete health in Para sport through pragmatic and accessible eHealth-based health promotion. It is a boundary-crossing project that will be in a real-world sport setting, including international researchers and sport organisations, and athletes training in different sport environments with different individual, geographical, social and economic ecosystems.

A limitation of the intervention could be the non-randomized control trial (RCT) design, as RCT design often is seen as the gold standard for evaluating an intervention.^19 32^ However, a crucial aspect in an RCT design is that the randomisation of the control group and intervention group is homogeneous and preferably matched, which is challenging in the Para sport population with a limited number of athletes, and athletes with various impairments and classifications participating in 28 different sports. Another crucial aspect is that RCT studies are most feasible when evaluating the effect of a single fixed intervention, for example, a specified dose of a drug.^32^ In contrast, injury and illness prevention in sport is complex, compromising multiple interacting components such as training background, general health, health literacy and coach and medical support as well as various outcomes such as acute injuries, overuse injuries, pain, general illnesses, overtraining and mental illness.^19^ Moreover, it has been shown that factors such as athlete behaviour and coaches’ perceptions of prevention may influence the outcomes of injury prevention,^5 8 12^ which are all factors that cannot be controlled within an RCT. In the Para sport population, interaction factors such as comorbidity from the impairment and various prerequisites may also interact with an intervention.

As many prevention studies in sport and exercise medicine fail to be implemented in a real-world sports context, it has been recommended to include the organisation, coaches and the target population itself in the development, implementation and evaluation,^6 17^ which also is supported by the Medical Research Council Guidelines, that suggest that a wider range and combination of research method advantageously could be used to improve interventions in real-world contexts.^18^ A strength of the current project is, therefore, that the implementation and evaluation of the implementation has been planned before the project starts. Also, the complex intervention design helps researchers and organisations to evaluate an intervention’s effectiveness, transferability and scalability in real-world contexts.^18 19^ A limitation is, however, that no participants from low-income countries have been involved in the development and implementation process. If this intervention is successful, it could, therefore, be suggested that a future RCT study can be conducted focusing on enrolling large multicentres of Para athletes from both low-income, middle-income and high-income countries, which also would allow us to randomise and match athletes.

## Data Availability

Data are available upon reasonable request. No data are available. This is a study protocol and hence no original data are available.
